# Gene Expression Profiling and Association with Prion-Related Lesions in the Medulla Oblongata of Symptomatic Natural Scrapie Animals

**DOI:** 10.1371/journal.pone.0019909

**Published:** 2011-05-24

**Authors:** Hicham Filali, Inmaculada Martin-Burriel, Frank Harders, Luis Varona, Jaber Lyahyai, Pilar Zaragoza, Martí Pumarola, Juan J. Badiola, Alex Bossers, Rosa Bolea

**Affiliations:** 1 Centro de Investigación en Encefalopatías y Enfermedades Transmisibles Emergentes, Facultad de Veterinaria, Universidad de Zaragoza, Zaragoza, Spain; 2 Laboratorio de Genética Bioquímica (LAGENBIO), Facultad de Veterinaria, Universidad de Zaragoza, Zaragoza, Spain; 3 Central Institute for Animal Disease Control (CIDC-Lelystad), Lelystad, the Netherlands; 4 Unidad de Genética Cuantitativa y Mejora Animal, Facultad de Veterinaria, Universidad de Zaragoza, Zaragoza, Spain; 5 PRIOCAT Laboratory, Centre de Recerca en Sanitat Animal (CReSA), UAB-IRTA, Campus de la Universitat Autònoma de Barcelona, Bellaterra, Barcelona, Spain; Creighton University, United States of America

## Abstract

The pathogenesis of natural scrapie and other prion diseases remains unclear. Examining transcriptome variations in infected versus control animals may highlight new genes potentially involved in some of the molecular mechanisms of prion-induced pathology. The aim of this work was to identify disease-associated alterations in the gene expression profiles of the caudal medulla oblongata (MO) in sheep presenting the symptomatic phase of natural scrapie. The gene expression patterns in the MO from 7 sheep that had been naturally infected with scrapie were compared with 6 controls using a Central Veterinary Institute (CVI) custom designed 4×44K microarray. The microarray consisted of a probe set on the previously sequenced ovine tissue library by CVI and was supplemented with all of the *Ovis aries* transcripts that are currently publicly available. Over 350 probe sets displayed greater than 2-fold changes in expression. We identified 148 genes from these probes, many of which encode proteins that are involved in the immune response, ion transport, cell adhesion, and transcription. Our results confirm previously published gene expression changes that were observed in murine models with induced scrapie. Moreover, we have identified new genes that exhibit differential expression in scrapie and could be involved in prion neuropathology. Finally, we have investigated the relationship between gene expression profiles and the appearance of the main scrapie-related lesions, including prion protein deposition, gliosis and spongiosis. In this context, the potential impacts of these gene expression changes in the MO on scrapie development are discussed.

## Introduction

Scrapie is a transmissible progressive neurodegenerative disease that occurs naturally in sheep and goats and constitutes one of the most widely studied models of transmissible spongiform encephalopathies (TSE) [1], a disease class that includes bovine spongiform encephalopathy (BSE) in cattle and human pathologies such as Creutzfeldt-Jakob disease and Kuru [1], [2], [3], [4]. A hallmark of all these diseases is the accumulation of an insoluble and protease-resistant isoform of the host-encoded prion protein (PrP), termed PrP^Sc^. According to the prion hypothesis, PrP^Sc^ is the principal component of the infectious particle, which is called a prion [5].

The neuropathology of prion diseases is characterized by the appearance and accumulation of PrP^Sc^ in the brain, spongiform degeneration, neuronal loss, and the activation of glial cells [6], [7], [8], [9], [10], [11], [12], [13].

Intensive research has aimed to investigate the relationship between the accumulation of prion protein PrP^Sc^, activation of microglia and astrocytes and the pathology of prion disease (e.g., neuronal loss) [12], [13], [14], [15]. Although several attempts have been made to understand the molecular events of these diseases [16], [17], [18], the precise molecular and cellular mechanisms that underlie prion disease pathogenesis, and even the role of PrP^C^ in host species, remain unknown.

The pathogenesis of scrapie is strongly influenced by genetics, and certain PrP polymorphisms are associated with individual susceptibility [19], [20], [21], [22]. However, genes other than PRNP (Prion Protein) are also thought to be involved in the pathogenesis of prion diseases [23], [24].

The identification of genes that are differentially expressed during prion infection may help identify novel risk genes and assist in the discovery of the abnormal intracellular or intercellular pathways that are responsible for the pathogenesis of prion diseases. Several functional genomics studies performed in experimental scrapie-infection animal models have indicated that several genes are misregulated in the advanced phase of the infection [25], [26], [27], [28], [29], [30]; these results are not surprising because the disease is preceded by neuronal loss and is usually fatal. Only a limited number of ovine genomics and expression analysis tools and data are available, and thus far, no study has focused on the “whole genome” expression (transcriptome) variation in natural scrapie sheep.

The objectives of the present study were to identify the differentially expressed genes in the brains of scrapie-symptomatic sheep using a CVI custom designed 4×44K microarray platform and to analyze any possible relationship between scrapie-related neuropathological changes and the transcriptional activities of the identified genes.

## Results

### Scrapie-related lesions

**Figure 1:**
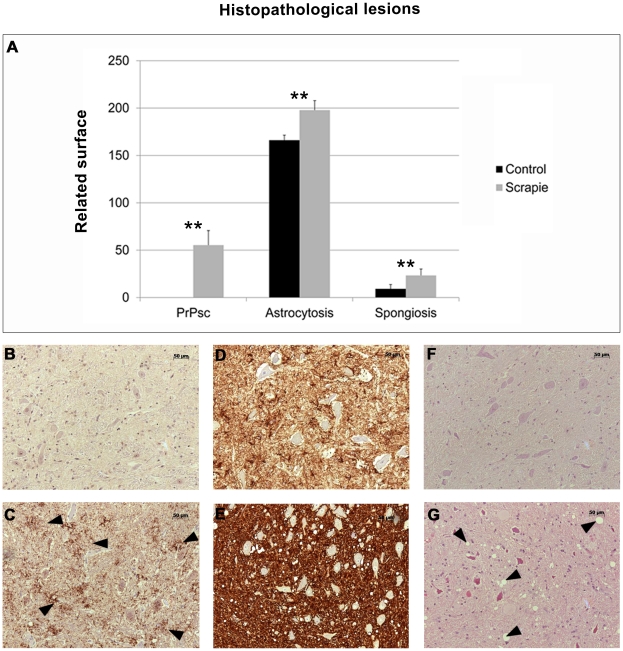
Quantitative values of PrP^Sc^ deposition, Glial fibrillary acidic protein expression as a marker for astrocytes and spongiform degeneration is shown as the mean ± standard error (A). Black bars: control sheep; grey bars: scrapie-infected sheep. Significant differences were determined using Student's *t* test (**P<0.01). PrPsc staining in control (B) and scrapie medulla oblongata sample (C). GFAP staining in control (D) and scrapie medulla oblongata sample (E). Haematoxilin/Eosine staining in control (F) and scrapie medulla oblongata sample (G).

The neuropathological features of scrapie were evaluated in the medulla oblongata tissue of 6 control and 7 clinical scrapie-infected sheep. Spongiosis, PrP^Sc^ deposition and GFAP immunoreactivity were consistent with the features of classical scrapie [31]. PrP^Sc^ deposition and spongiosis were only detected in the affected animals (Figure 1). Particular medullary nuclei in the obex, such as the nucleus dorsal motor of the vagus, the spinal tract of the trigeminal nerve and the solitary tract nucleus, were severely affected in the infected group. The low level of spongiosis observed in the control animals was an artifact due to the method of analysis (ImageJ). Even with the high variability observed in the scrapie group, the differences between the groups were statistically significant (*P*<0.01).

A generalized increase in the expression of the astroglial marker glial fibrillary acidic protein (GFAP) was observed in the brains of the scrapie-affected sheep (*P*<0.01). Hyperplasia and hypertrophy of the stellate GFAP-positive cells, consistent with reactive astrogliosis, was observed in the medulla oblongatas of the affected sheep.

### Identification of genes in the medulla oblongata that are differentially expressed in natural scrapie

Transcriptome profiles in the medullae of sheep affected by scrapie and controls were obtained using the Central Veterinary Institute (CVI) custom 4×44K microarray, containing a custom eArray probe design from the earlier sequenced ovine tissue cDNA libraries supplemented with all of the currently available transcripts from the NCBI/EBI databases and with probes available from the Agilent ovine catalog. A total of 300 probe sets displayed statistically significant differences between the control and scrapie groups that were equal to or greater than a 2-fold change. Genes from *Ovis aries* are relatively poorly annotated, but after BLAST searches to publicly available databases, it was possible to identify a set of 148 known genes (Table S1) from the complete set of 300 differentially expressed genes. The microarray data have been deposited in the array express and are accessible through accession no. E-MTAB-532. To determine the gene ontology (GO) categories of the deregulated genes in the scrapie condition, we used DAVID Bioinformatics Resources 2008 [32], [33] (NIAID/NIH, USA). Based on the GO analysis, 93 genes had known functions; 40 genes were upregulated (43%), and 53 genes were downregulated (57%). The functional group with the highest number of regulated genes was the ion binding-related genes (15 genes), followed by the nucleotide binding-related genes (11 genes), the structural molecule activity genes (8 genes), the immune system-related genes (7 genes) and the ion transport-related genes (5 genes) (Table S1).

**Figure 2:**
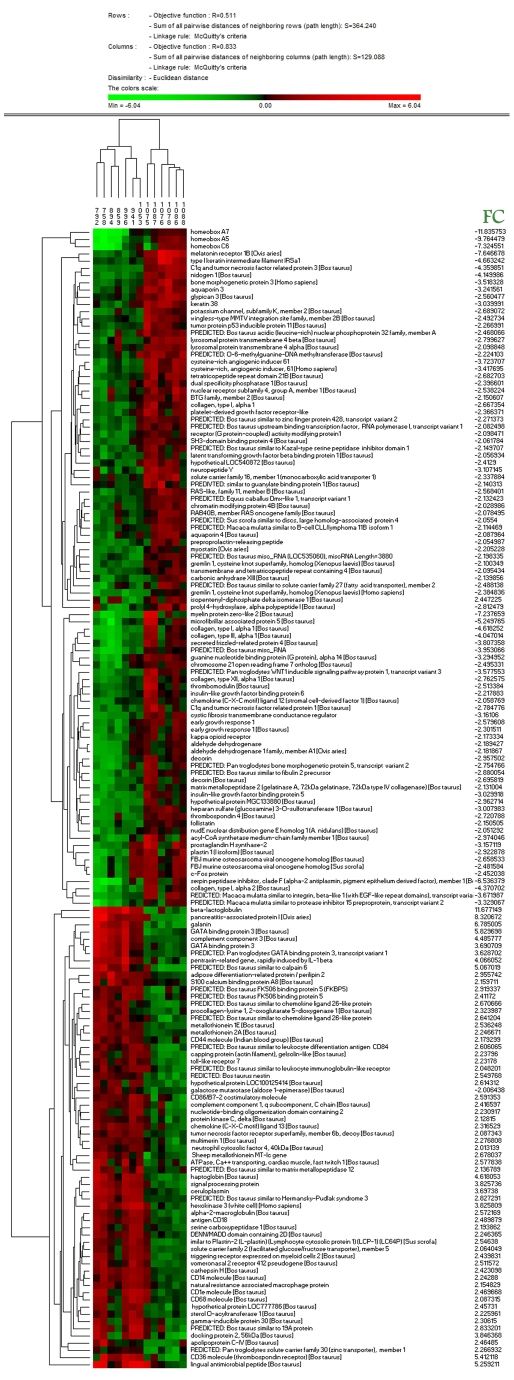
Condition trees of clustering analysis. The hierarchical cluster analysis (Euclidean distance clustering algorithm) was performed using PermutMatrix [100] and 148 genes that differed significantly and had a known function. Each colored bar represents a gene. The color represents the level of expression, and the sample information is listed across the top. The gene names are indicated. Note the distinct patterns of altered gene expression between the positive and control animals.

The significance of each GO term was also evaluated using an enrichment analysis, which calculates the significance of each cluster based on the proportion of differentially expressed genes that contributes to the respective cluster. The only significantly (P<0.05) deregulated cellular component GO term was the one corresponding with extracellular region proteins, including 24 deregulated genes (15.4% of the total modified known genes) with P = 1.0×10^−9^, of which 11 are extracellular matrix components (P = 1.7×10^−7^). Six GO terms related to molecular function were significantly deregulated: calcium ion binding (9 genes, P = 9.8×10^−3^), growth factor binding (4 genes, P = 3.0×10^−3^), extracellular matrix structural constituent (3 genes, 6.3×10^−3^), SMAD binding (3 genes, 3.7×10^−3^), carboxylic acid binding (3 genes, 4.8×10^−2^) and platelet derived growth factor binding (2 genes, P = 2.8×10^−2^). After clustering analysis, the animals studied were grouped according to their disease condition (Figure 2).

### Validation of gene expression profiling by quantitative RT-PCR

To confirm the results of the microarray, we carried out quantitative RT-PCR using SYBR Green assays on a selected number of targets. For validation, we chose three upregulated (calpain 6 [*CAPN6*], galanin 1 [*GALA1*] and pancreatitis associated protein 1[*PAP1*]) and three downregulated (collagen 1 alpha 2 [*COL1A2*], collagen 3 alpha 1 [*COL3A1*] and melatonin receptor 1b [*MTNR1B*]) genes that had previously been reported to be associated with prion and other neurodegenerative diseases [34], [35], [36], [37], [38], [39], [40].

**Figure 3:**
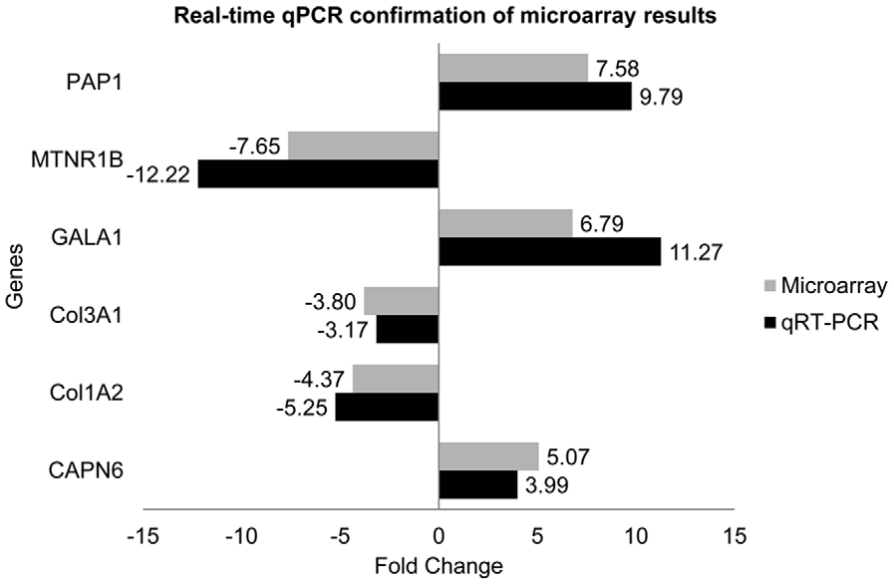
Relative mRNA levels. Indicated by fold change versus controls, the expression levels of the selected genes analyzed by microarray (grey bar) and quantitative RT-PCR (black bar) in the scrapie medulla oblongata are shown.

The quantitative RT-PCR analyses confirmed the microarray expression results (Figure 3). Differences between the control and scrapie groups were statistically significant for each of the 6 genes analyzed (P<0.05).

### Identification of neuropathology-related genes

**Figure 4:**
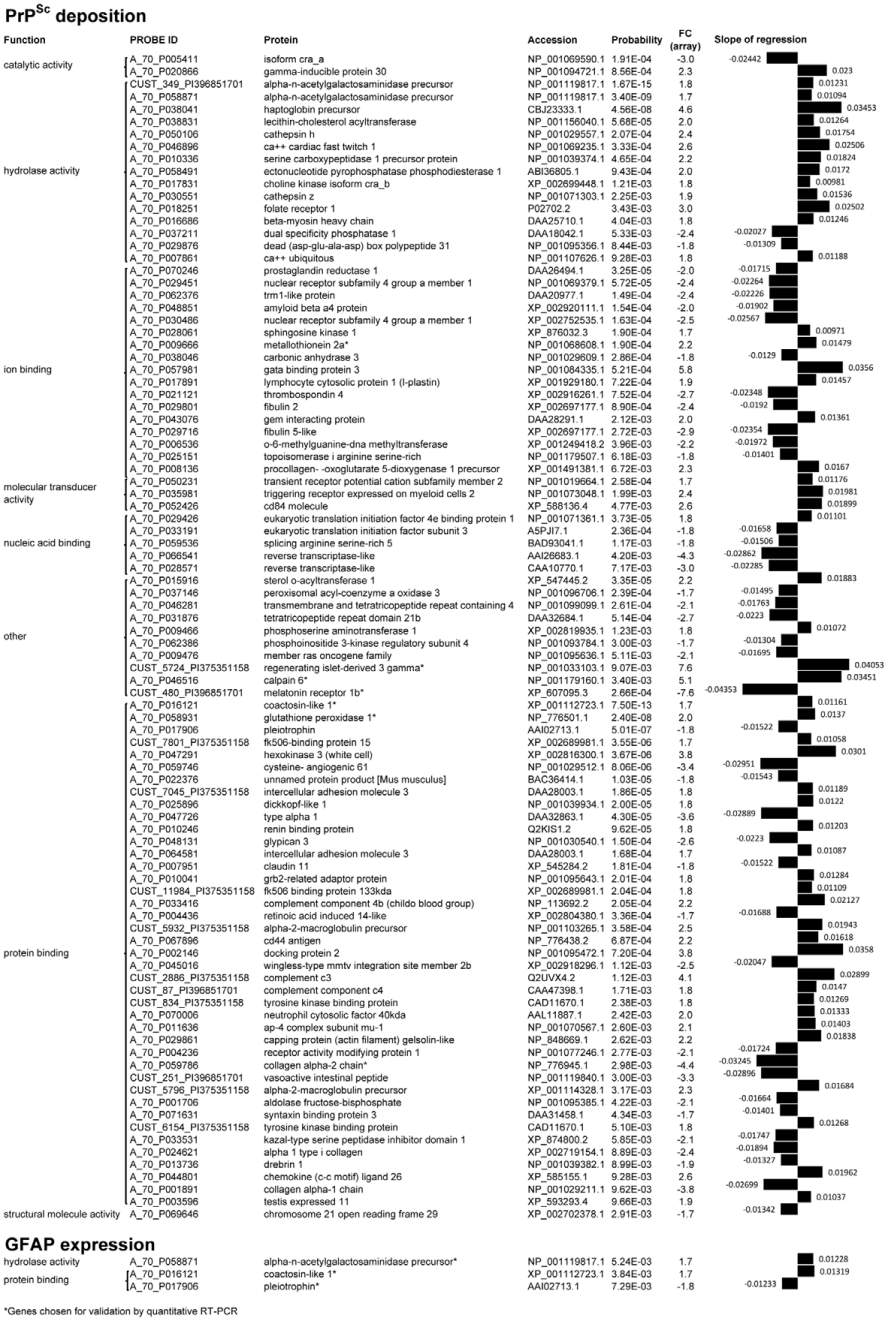
Relationship between gene expression profiles and scrapie histopathological lesions. A selection of genes whose expression was significantly related to PrP^sc^ deposition and glial fibrillary acidic protein expression grouped by their function is shown. Probe ID, accession numbers of proteins encoded by these genes and probability of the slope of regression between histopathological lesions and gene expression obtained using a Mixed Model approach are shown.

To identify the relationship between gene expression profiles and scrapie neuropathology, a Mixed Model approach was performed using the Gene Expression Analysis with Mixed Models (GEAMM) software [41]. The program analyzes the continuous effect of scrapie related lesions, measured as PrP^Sc^ deposition, GFAP immunostaining and spongiosis, on individual gene expression. GEAMM v1.4 gives the probability of the regression coefficient obtained between gene expression and the scrapie lesions. As the reliability of microarray experiments is optimal for fold changes values higher than 1.7, we developed this analysis with genes whose expression was modified at a level equal to or higher than this proportion. We identified 357 probe sets whose expression was related to PrP^Sc^ deposition and 12 related to astrocytosis. Any probe was related with spongiosis. After BLAST searching, these probes corresponded to 98 genes, of which 94 could be linked to prion deposition and 4 could be linked to astrocytosis. Gene ontology analysis revealed genes mainly related to protein and ion binding and hydrolase activity in the association study. A list of known genes whose expression is highly correlated with PrP^Sc^ deposition and GFAP expression is indicated in Figure 4.

### Validation of neuropathology-associated genes

In addition to the previously described 6 genes that were used to validate the array results, 5 additional genes were chosen for validation using quantitative RT-PCR, of which two, glutathione peroxidase 1 [*GPX1*] and metallothionein 2A [*MT2A*], were associated with prion deposition, one, pleiotrophin [*PTN*], was associated with GFAP expression, and two, coactosin-like 1 [*COTL1*] and N-acetylgalactosaminidase alpha [*NAGA*], were associated with prion deposition and GFAP expression. The genes were selected based on their known function in the brain or their association with other neurodegenerative diseases. Linear regression was calculated between the normalized expression values of these genes and prion deposition, GFAP immunoreactivity and spongiosis scores/rates. Four of the 6 genes previously analyzed for array validation (*COL1A2*, *MTNR1B*, *PAP1* and *CPN6*) also displayed significant associations with prion deposition. Therefore, these genes were also included in the validation regression analysis. Table 1 indicates the posterior probability values of linear regression between the chosen genes and the neuropathology. The previously established associations were confirmed in 7 of the 9 analyzed genes.

**Table 1:** Confirmation of the association results for 9 genes using quantitative RT-PCR and linear regression.

PRION RELATED GENES				
Gene	b		FC	
	Microarray	qRT-PCR	Microarray	qRT-PCR
**CAPN6**	0.03451**	0.008486*	5.1**	3.99*
**COLIA2**	−0.0435***	−0.007667*	−4.4**	−5.25**
**COTL1**	0.0116***	0.00913**	1.7**	1.64*
**GPX1**	0.0137***	0.00967**	2.0**	2.14**
**MT2A**	0.01479***	0.00950***	2.3**	1.91***
**MTNR1B**	−0.0325***	−0.009857*	−7.7**	−12.22*
**PAP1**	0.04053**	0.007388	7.6**	9.79*
**NAGA**	0,01094***	0.01496**	1.8**	1.66**

GLIOSIS RELATED GENES				
Gene	b		FC	
	Microarray	qRT-PCR	Microarray	qRT-PCR
**COTL1**	0.01319**	0.00913**	1.7**	1.64*
**NAGA**	0.01398**	0.01496**	1.8**	1.66**
**PTN**	−0.01233**	−0.0090	−1.8**	−1.52**

Slope values (b) and association probabilities (* P<0.05; ** P<0.01, *** P<0.001) for microarray and RT-PCR data. Gene expression changes in scrapie medullae indicated by the fold change (FC) obtained with microarray (grey bar) and RT-PCR data (black bar) and their statistical significance (* P<0.05; ** P<0.01, *** P<0.001).

## Discussion

The neuropathology of prion disease is characterized by an accumulation of the pathological form of the prion protein, spongiform changes and reactive gliosis [42]. Although the lesion pattern of these diseases has been described in great depth, the precise mechanisms regulating these processes remain unknown. Genomic approaches show an extraordinary potential to uncover the molecular basis of complex mechanisms and to discover new biomarkers. A number of genomic analyses of brain tissue from rodent-adapted models of prion diseases (including CJD, scrapie and BSE) have been performed [25], [26], [27], [28], [29], [30]. However, there are fewer studies focused on the study of mRNA profiles in natural human CJD [43], bovine BSE [44] or ovine scrapie [45]. We present here for the first time the genomic expression variations of natural scrapie, using samples from natural symptomatic scrapie-affected sheep, and the correlation with scrapie-associated neuropathology.

To identify the differentially expressed genes in natural scrapie, we applied a global analysis of the overall transcriptome response in caudal medulla oblongata tissue from natural scrapie-affected sheep in a terminal stage of the clinical disease. This analysis was performed using the CVI custom 4×44K oligo-DNA microarray platform containing 13 k 60-mer oligos representing previously sequenced clones from a custom normalized cDNA library of sheep Peyer's Patch, tonsil and brain, supplemented with all publicly available transcripts from NCBI/EBI databases (unpublished data).

Statistical analysis identified 300 significantly changed probes having that met the threshold of a 2-fold change of expression compared to controls. These 300 probes could be linked to 93 genes with a known function based on gene ontology (GO) analysis. In accordance with previous reports [26], [46], the genes identified in this study were included in the following major groups: secreted extracellular proteins, lysosomal proteases, defense and immune response-related proteins and signal transduction-related genes.

The following four genes were previously described in other studies related to gene expression alteration in brains of murine scrapie: insulin-like growth factor binding protein 5, early growth response 1, ATPase Ca++ transporting cardiac muscle and aldehyde dehydrogenase [26], [30], [47]. The directions of these gene expression changes were in accordance with the changes observed in our study.

In previous studies, the ApoE protein has been proposed as a biomarker for prion diseases [48], [49], [50], [51] in addition to its role in the Alzheimer's disease pathway [52], [53]. Several studies have also reported the involvement of many members of the apolipoprotein gene family (*ApoA1*, *ApoA4*, *ApoC1*, *ApoC2*, *ApoC3* and *ApoD*) in transmissible spongiform encephalopathies [26], [30], [54], [55]. Although the expression of these genes was not modified in our study, the *ApoC4* (apolipoprotein C-IV) gene was upregulated in our scrapie samples. *ApoC4* is normally primarily expressed in the liver and is involved in lipoprotein metabolism. The role of *ApoC4* in the brain remains unclear, but it is thought to be involved in nerve growth, regeneration and neuronal repair. Our results indicate that this gene, a member of a family previously associated with TSEs, may also participate in scrapie-associated neuropathology and/or repair.

The microarray data also indicated a 5-fold increase in the expression of a gene similar to bovine calpain 6. This overexpression was confirmed by quantitative RT-PCR. Calpains are a family of calcium-activated intracellular cysteine proteases that are involved in many physiological events, such as proteolysis, apoptotic cell death, and necrosis [56], [57]. In agreement with our results, perturbations in the activity of other members of the calpain family (e.g., calpain 1) have been associated with the neuropathological processes contributing to Alzheimer's disease [58], [59].

Galanin (*GALA1*) overexpression has also been reported in Alzheimer's disease [34], [35], and it has been demonstrated that an increase of this neuropeptide impairs cognitive function [39]. In our study, the expression of *GALA1* showed 7- and 11-fold increases according to the microarray and qRT-PCR analyses, respectively. This is the first time that the overexpression of this gene has been associated with prion diseases, suggesting a possible role of *GALA1* in some of the behavior alterations reported in scrapie [60].

The pancreatitis-association protein I (*PAP1*) gene displayed high upregulation in the medullae of scrapie-affected sheep, according to both microarray and qRT-PCR data. Although PAP1 is an inflammatory protein that is specifically overexpressed in pancreatitis [61], it is elevated in the ileal Peyer's patch of lambs during the early phase of scrapie infection [62]. Moreover, the intervention of PAP1 in very early stages of Alzheimer's disease has been reported [38]. Our results confirmed the participation of PAP1 in scrapie pathogenesis, not only in the early phases of the diseases but also in the clinical stage.

The expression of the melatonin receptor 1B (*MTNR1B*) gene was highly downregulated in the medullae of affected animals. This finding is in agreement with the neuroprotective activities of melatonin described in Alzheimer's disease [63], [64]. Consequently, the decrease in *MTNR1B* gene expression could contribute to the brain damage observed in scrapie-affected brainstems.

Variations in the gene expression of different collagens have also been reported in Alzheimer's disease. Although increased levels of collagen XXV promote an Alzheimer's-like disease in mice [65], the upregulated expression of collagen VI in neurons of Alzheimer's disease brains may represent a protective mechanism [66]. In our results, three types of collagen (*COL1A2*, *COL3A1* and *COL12A1*) exhibited downregulation in sheep with natural scrapie and might be related to the loss of their neuroprotective role.

Changes in the extracellular matrix (ECM) have been previously described in prion diseases [15], [67], but until now, specific studies based on collagen changes in the ECM have not been performed. Overall, collagens are not abundant in the ECM of the brain, but they are present in the vascular basement membrane [68]. The area studied includes a circumventricular organ, the area postrema, which is a structure rich in different collagen types and one of the entrances of the prion to the brain [69]. Further studies will be necessary to clarify the effects of prion infection on the synthesis of collagens.

Most microarrays reports have focused on comparisons between healthy and scrapie tissues [28], [29], [30]. However, when we analyze tissues from animals naturally infected with scrapie, the degree of lesion for each individual can be very different, even for animals included in the same group (healthy or scrapie affected). These differences could influence the individual expression profiles. In the present study, we aimed to investigate the effect of prion-related lesions on gene expression, which would contribute to the knowledge of the molecular mechanisms of scrapie neuropathology. Immunohistochemical and hematoxylin-eosin image quantification of PrP^Sc^ deposition, GFAP immunostaining and spongiosis could be considered continuous effects. We used the GAMM v1.4 software [41] to perform a Mixed Model Analysis using a Bayesian approach that allowed the establishment of associations between gene expression data and prion deposition, gliosis and spongiosis. The spongiosis, PrP^Sc^ deposits and generalized reactive gliosis observed in the medulla oblongatas of scrapie-affected animals in the present study were in accordance with previous descriptions of classical scrapie [31], [70], [71], [72], [73] and support the hypothesis that PrP^Sc^ deposition elicits a responsive glial cell proliferation and the appearance of spongiotic lesions [15], [74], [75], [76]. The increase of GFAP in affected animals was in accordance with the microarray experiment results, in which the GFAP gene was upregulated in scrapie medullae with a 1.7-fold change (data not shown in Table S1 because only genes with a FC>2 are represented). The mechanisms associated with the expression of GFAP in scrapie-affected medullae, along with the molecules involved in prion deposition or spongiform degeneration, remain unclear. The association between gene expression changes and these processes could contribute to the knowledge of their molecular pathways.

In contrast to the scrapie vs. healthy comparison study, most of the genes that displayed highly significant associations with scrapie neuropathological lesions are new candidate markers and have not been previously linked to prion diseases. This method allowed us to identify genes that could be related to prion-specific processes without the need for a high and significant difference between the controls and the infected animals. The association study revealed a high number of genes that are associated with prion deposition in the medulla oblongata. From these, the genes that showed the highest association probability were mainly involved in protein, nucleic acid and ion binding and hydrolase activity. Coactosin-like protein (*CLP*) shares significant homology with coactosin, an F-actin binding protein from *Dictyostelium discoideum* [77]. *CLP* mRNA is widely distributed throughout the tissue and is expressed at high levels in the placenta, lung, kidney, and peripheral blood leukocytes and at low levels in the brain, liver and pancreas [78]. Altered levels of structural proteins have been observed in neurological disorders using proteomic assays [79], [80], [81]. For example, increases of coactosin-like protein 1 (*COTL1*) have been reported in a proteomic analysis in Parkinson's disease that was proposed to be associated with a structural reorganization of parkinsonian substantia nigra [82]. In agreement with this, we identified a positive association between the expression of this gene and the levels of prion deposition and GFAP immunoreactivity, along with a significant overexpression in scrapie medullae (FC: 1.76) that suggests a role for this structural protein in different neuropathological processes.

Oxidative stress has been proposed to play an important role in the pathogenesis of prion disease [83]. PrP^C^ plays an important role in anti-oxidative defense, and its deficiency increases susceptibility to oxidative stress [84]. Glutathione peroxidase is one of the major antioxidant enzymes in the detoxification of hydrogen peroxide [85], [86]. The brain is considered to have lower *GPx-1* activity compared with other tissues [85]. This enzyme is abundant in activated microglia and is present at low levels in most neurons [85]. In vitro studies have indicated that the lack of *GPx1* enhances the toxicity of the amyloid beta-peptide [87]. *GPx-1* positive microglia are increased in Parkinson's disease and dementia with Lewy bodies, and it has been proposed that *GPx-1* can participate in a cellular process to enzymatically degrade concentric Lewy bodies [88]. In accordance with that finding, we observed a positive association between the expression of this gene and prion deposition and a significant overexpression of *GPx-1* in scrapie medullae (FC: 2.03). The role of this protein in prion diseases has not been previously investigated. Further analysis, including immunohistochemical determination of *GPx-1* in scrapie brains or in vitro studies, would be necessary to elucidate a possible neuroprotective role of this enzyme in prion diseases.

Metallothioneins are a group of metal-binding proteins found in a variety of eukaryotic and prokaryotic species [89], [90]. The mouse MT I and II genes are coordinately regulated by metals, glucocorticoids and inflammatory stress signals [91]. It has been shown that the gene coding for MT II is overexpressed in the brains of scrapie-infected hamsters [92], [93], mice [94] and BSE-affected medulla oblongata [90]. Immunoreactivity for MT II has been detected consistently in the astrocytes of BSE cases [90]. The labeling for this protein has also been reported in natural scrapie [15], particularly in the brain stem and thalamus, not only in astrocytes and amoeboid-shaped glial cells but also in neuronal perykaria. In accordance with this finding, we observed a significant overexpression of the metallothionein 2A (MT2A) gene in scrapie medullae (Table 1), which was positively associated with prion deposition but not with GFAP immunostaining.

N-acetylgalactosaminidase alpha (NAGA) cleaves alpha-N-acetylgalactosaminyl moieties from glycoconjugates. Mutations in NAGA have been identified as the cause of Kanzaki disease, which may involve neurologic complications in the CNS and peripheral nervous system [95]. In the present study, we observed a slight but significant increase of NAGA expression in scrapie medullae and a positive association between the expression of this gene and both prion deposition and astrogliosis. To our knowledge, this is the first time that modifications in this gene have been reported in prion diseases.

In conclusion, our genomic analysis allowed the identification of new genes involved in the neuropathology of natural scrapie in the medulla oblongata and the confirmation of genes that were previously described as biomarkers of other neurodegenerative diseases, such as Alzheimer's and Creutzfeldt-Jakob disease in humans. Likewise, as recently reported [96], [97], our findings confirm the depth of the relationship between scrapie and other neurodegenerative diseases. In addition, our association analysis contributes to the knowledge of the molecular mechanisms underlying the pathogenesis of prion diseases. Further studies of the cellular localization of the proteins coded by these differentially regulated genes are necessary to establish the specific and thus far unknown roles of these markers in TSE pathogenesis. In addition, the study of the expression of identified genes in other brain areas, as well as in preclinical scrapie infected animals, could contribute to the knowledge of their role in the disease.

## Materials and Methods

### Ethics Statement

This study was carried out in strict accordance with the recommendations for the care and use of experimental animals of the University of Zaragoza, in accordance with law (R.D. 1201/2005). The protocol was approved by its Committee on the Ethics of Animal Experiments (Permit Number: PI02/08).

### Animals

A total of 13 Rasa Aragonesa female sheep (aged 3–5 years) were included in this study. Seven of the animals exhibited clinical signs of scrapie, and the diagnoses were made by third eyelid biopsies [60] and confirmed using the rapid test (TeEsE, Bio-Rad) and immunohistochemistry to detect PrP^Sc^ using the 6H4 monoclonal antibody [72]. We would like to emphasize that all of the sheep (n = 7) were in the terminal stage of the disease. This characterization was carried out following previously reported criteria [60], taking into account the presence of the clinical signs associated with the disease. Moreover, all of the animals belonged to flocks that had been previously characterized as scrapie-affected flocks and were located in different geographical areas. The animals were genotyped for PRNP polymorphisms as previously reported [98], and the sheep chosen for this study displayed the ARQ/ARQ genotype, which is the most susceptible genotype in this ovine breed [98]. The control animals (n = 6) were all female, belonged to the same breed, were a similar age (3 to 5 years old) and had identical PRNP genotypes (ARQ/ARQ). They were selected from flocks located in areas free of scrapie. The presence of prion protein was confirmed by immunohistochemical methods and western blotting [72].

### Tissue collection and RNA isolation

Animals were sacrificed by intravenous injection of sodium pentobarbital and exsanguination, and the necropsy was performed immediately. Physical examination of the scrapie and control animals did not reveal any other pathological signs. The samples were rapidly preserved and processed according to established guidelines regarding safety. Because the lesion pattern in scrapie is bilateral, one half of the caudal medulla oblongata, including the obex, was snap-frozen in liquid nitrogen prior to long-term storage at −80°C until RNA extraction. The other half was formalin-fixed and paraffin-embedded for further histopathological analysis. Total RNA was isolated from a tissuemizer disrupted medulla oblongata in duplicate using TRIzol® (Invitrogen AG), followed by a phenol and chloroform extraction and subjected to a purification step with the NucleoSpin®RNA clean-up kit RNAII (Macherey-Nagel GmbH & Co. KG). The quality of the total RNA was assessed using the RNA 6000 Nano Assay kit and the 2100 Bioanalyzer (Agilent Technologies). The Agilent 2100 Expert software was used to estimate the RNA integrity number (RIN) index for each sample. The RIN provides a numerical assessment of the integrity of RNA that facilitates the standardization of the quality interpretation. Only high quality RNA samples with an RIN number equal to or higher than 7 were further processed for microarray analysis.

### Histology and prion immunohistochemical detection

A histopathological study of the medulla oblongata at the level of the obex was performed in HE-stained slices (one from each individual control and each positive animal).

Immunohistochemical (IHC) studies were performed on adjacent sections. Positive and negative controls (omission of primary antibodies in the control and scrapie slides) were performed for every antibody.

Prion protein detection was performed following pretreatment as previously described [99]. Briefly, sections were pretreated with 98% formic acid and hydrated, autoclaving to enhance antigen retrieval. After proteinase K digestion (Roche, 4 g/ml), the sections were incubated with blocking reagent (DAKO) for 10 min to block endogenous peroxidase activity. Next, sections were incubated with the monoclonal primary antibody L42 (R-Biopharm, dilution 1∶500) at RT for 30 min. Sections were processed with endogenous peroxidase blocking. The enzyme-conjugated polymer Envision (DAKO, 30 min) was used as the visualization system and DAB (DAKO, 10 min) as the chromogen. Sections were counterstained with hematoxylin.

Astrogliosis was evaluated based on glial fibrillary acidic protein (GFAP) immunostaining, as previously described [15], [16]. Briefly, after heat-induced epitope retrieval pretreatment with citrate buffer (pH 6.0), the sections were incubated for 1 h at RT with the rabbit polyclonal anti- GFAP antibody (DAKO, dilution 1∶400). In routine immunoreactions, omission of the primary antibodies in control and scrapie slides served as negative controls.

The preparations were examined with a Zeiss Axioskop 40 optical microscope (Carl Zeiss AG) and a 40× magnification objective lens (Carl Zeiss AG). The images were captured with a digital camera (AxioCam MRc5, Zeiss AG) that was coupled to the microscope and a computer and were analyzed using the ImageJ 1.4.3.67 image-analysis software package (Psion Image, NIH) to determine the areas occupied by PrP^Sc^ deposition, astrogliosis and spongiosis. For the evaluation of IHC and HE slides, captured images were opened in NIH Image/ImageJ to evaluate the indices of positivity using the area method. The total area occupied by brown markers (PrP and GFAP) or by white spaces (spongiosis) was estimated by setting a “threshold” using the thresholding tool for selection of these areas and the positive IHC/HE index for that image was calculated. Significant differences between the control and scrapie groups were detected using the Student's *t* test.

### Custom sheep oligo-DNA microarray

CVI custom 4×44K microarrays were used. They contained custom eArray designed 60-mer probes on previously sequenced normalized and subtracted cDNA libraries of ovine Peyers Patch, obex and tonsil, supplemented by the publicly available *Ovis Aries* transcripts from NCBI/EBI databases and by the Agilent *Ovis Aries* transcript catalog. All of the arrays were printed using Sureprint technology (Agilent Technologies).

### Preparation of labeled cDNA and microarray hybridization

All of the procedures for the preparation of labeled cRNA probes and subsequent Genechip hybridizations were performed according to the Agilent Technologies One-Color Microarray-Based Gene Expression Analysis guidelines (http://www.home.agilent.com). First, cDNA was synthesized using 1 µg total RNA as the template and T7 Promoter Primer of Agilent One-Color RNA Spike-In (Quick Amp Kit, One-Color, Agilent Technologies). cDNA was then transcribed and labeled using T7 RNA Polymerase and cyanine 3-CTP. Finally, labeled cRNAs were cleaned up using Qiagen's RNeasy mini spin columns.

The samples were then hybridized to CVI-Agilent custom 4×44K chips. All of the hybridizations were carried out for 17 h at 65°C and 6 rpm. The chips were then washed and incubated with wash buffers following the manufacturer's protocol and scanned using the GenePix 4200AL Scanner (Axon Instruments) in conjunction with GenePix Pro 6.0 software.

Hybridizations of each sample were performed in duplicate, resulting in 14 microarrays for clinical scrapie animals and 12 for negative control animals.

### Microarray Data Analysis

The hybridization data were extracted with the feature extraction 9.5.3.1 image analysis application (Agilent Technologies) before processing with GeneSpring GX 10.0.2 (Agilent Technologies). Intensity values of the chips were normalized using the 75th percentile method and the expression values were calculated. The global medulla oblongata gene expression profiles from clinical scrapie infected animals were compared to the negative controls. Furthermore, only genes with a Student's *t* test *P*-value≤0.05 and a 2-fold change as the lower limit were selected. These genes were clustered by their Euclidean distance coefficient using the Permutmatrix software [100]. A BLAST search of the GenBank database was performed to identify the genes that were similar to the differentially expressed probes. Molecular functions of the genes were classified according to Gene Ontology (GO), using on-line functional annotation of DAVID Bioinformatics Resources 2008 [32], [33] (NIAID/NIH, USA).

### Relationship between neuropathology and gene expression

The relationship between neuropathological lesions and gene expression was performed using a Mixed Model Analysis under a Bayesian approach by the Gene Expression Analysis with Mixed Models (GEAMM) software [41].

The statistical model assumed the following Bayesian likelihood of logarithm of gene expression data provided by the oligo-DNA microarray:
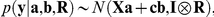
where *a* is the array effect and *b* is the vector regression slope associated with the numerical valuation of the neuropathological changes (*c*: prion deposition, spongiosis or astrogliosis). Moreover, *X* is the incidence matrix that relates the array effects to the logarithm of gene expression data (*y*). Finally, *R* is the matrix of residual (co)variances with probe-specific residual variance and null residual covariances. Prior distributions were assumed to be flat for *a*, *b* and *R*. A more detailed description of the statistical procedure was presented by Casellas et al. (2008) [41].

The Bayesian analysis was performed using a Gibbs sampler approach [101] with a single chain of 500,000 iterations after discarding the first 50,000. The results with a posterior probability below 0.01 for a regression slope associated with a neuropathological lesion greater (or lower) than zero were selected.

### Real-time quantitative PCR

We performed quantitative real-time RT-PCR (qRT-PCR) to confirm the expression of 6 genes that were expressed at a level higher or lower than three times in the scrapie group compared to controls in the oligo-DNA microarray expression analysis, and 8 genes that displayed the highest significance in the Mixed Model Analysis. The PCR primer sequences used for quantification of the genes encoding *CAPN6* (calpain 6), *COL1A2* (collagen Ia), *COL3A1* (collagen IIIa), *GALA1* (galanin 1), *MTNR1B* (melatonin receptor 1B), *PAP1* (pancreatitis associated protein 1), *COTL1* (coactosin-like 1), *GPX1* (glutathione peroxidase 1), *MT2A* (metallothionein 2A), *PTN* (pleiotrophin) and *NAGA* (N-acetylgalactosaminidase, alpha) are indicated by Table 2. The quantitative PCR assays were designed using Primer Express 2.0 software (Applied Biosystems) to select appropriate primer sequences from known sheep or bovine sequences. Whenever possible, the exon–exon border was considered when designing the primers to prevent the amplification of genomic DNA in the PCR reaction. Complementary DNA (cDNA) was synthesized from 1 µg of each RNA using random hexamer primers with the Superscript First Standard Synthesis System for RT-PCR (Invitrogen). Retrotranscription with and without enzyme was performed to confirm the elimination of any remaining DNA.

**Table 2:** Genes analyzed by quantitative real-time PCR.

	Gene	Primer sequence	Size (bp)	Accession number
Genes to analyse	CAPN6	F: TGCAGAACCCCCAGTACATCTT	81	NM_001192231.1**
		R: TGCGCAGGTCCTTTTGCT		
	COL1A2	F: GCCTAGCAACATGCCAATCCT	72	NM_174520.2**
		R: CGCGTGGTCCTCTATCTCCA		
	COL3A1	F: CGACCAAGAATTAGACTGCCCC	82	NM_001076831.1**
		R: GGGAGCTGTTGGAGGCTGT		
	COTL1	F: CAAGTTCGCCCTCATCACATG	104	NM_001046593.1**
		R: CGAAATTCTGCACCACCTCCT		
	GALA1	F: CTTCTCGGACCACATGCCAT	97	EF192581.1*
		R: GCCGGGCTTCGTCTTCAG		
	GPX1	F: GGGCATCAGGAAAACGCC	81	NM_174076.3**
		R: GTTGGGCTCGAACCCGC		
	MT2A	F: TCCTGCAGCTGTGCTGGCT	65	NM_001040492.1**
		R: CAGCTCTTCTTGCAGGAGGGAC		
	MTNR1B	F: TCCGGAACGCAGGTAACCT	95	NM_001130938.1*
		R: GAAGATGGCCGCAAGGGT		
	NAGA	F: TCTCAAGGAGAAATCCCACATTG	71	NM_001046349.1**
		R: AGAAGACGATGGCGCTGG		
	PAP1	F: AGCTGCCTCCACTCCACATG	81	NM_001038014.1*
		R: TCAGGCAGGAGAGCAGCATC		
	PTN	F: TCCAAAATGCAGACTCCACAGTAC	51	NM_173955.1**
		R: AGCTGCAAATTTTCGACGTTG		

Primers (F: Forward and R: Reverse) used for gene amplification, amplicon size and GenBank accession numbers of the sequences used for primer design.

*Ovine cDNA.

**Bovine cDNA.

PCR reactions were performed using SYBR® Green (PE Applied Biosystems) assays. PCR amplification was performed in an ABIPrism fast 7500 Sequence Detection System (PE Applied Biosystems). All of the RT-PCR reactions were run in triplicate with total reaction volumes of 10 µl, using 10–20 ng of cDNA as template and 300 nM as the final concentration of primers. Universal conditions were used, with an initial 10 min activation and denaturation step at 95°C, followed by 40 cycles of 15 s at 95°C and 30 s at 60°C. The baseline and threshold for Ct calculation were set automatically with the ABI-Prism 7500 software Version 2.0.1. The levels of gene expression were determined using the comparative Ct method.

To improve the normalization accuracy, the geometric mean of three housekeeping genes was used to calculate the normalization factor (NF), which was used to normalize the expression level of each gene in each sample [102]. The NF was calculated from the GAPDH, G6PDH and RPL32 expression data. These genes are the three most stable reference housekeeping genes in sheep medulla oblongata and have been used as internal references for expression studies in scrapie [103]. Primers and PCR conditions for the amplification of these housekeeping genes have been previously described [103], [104].

Quantitative results obtained from qRT-PCR assays are expressed as fold change. Student's *t* test analyses were used to determine whether the differences observed between groups were statistically significant (*P*<0.05) for the 6 differentially expressed genes, and a linear regression was performed to confirm the relationship observed in the 8 genes chosen after Mixed Model Analysis.

## Supporting Information

Table S1 List of differentially expressed genes in natural Scrapie medulla oblongata and their associated functions based on GO analysis. Only those genes with FC>2 and a known GO term are shown. References for differentially expressed genes previously reported in other TSEs. *Genes chosen for validation by quantitative RT-PCR.
(DOC)
