# Supplementary material for: Gene Expression Profiling and Association with Prion-Related Lesions in the Medulla Oblongata of Symptomatic Natural Scrapie Animals
Source: PLoS One. 2011 May 24;6(5):e19909. doi: 10.1371/journal.pone.0019909 (PMC3101219; doi:10.1371/journal.pone.0019909)
Supplement: Table S1 — List of differentially expressed genes in natural Scrapie medulla oblongata and their associated functions based on GO analysis. Only those genes with FC>2 and a known GO term are shown. References for differentially expressed genes previously reported in other TSEs. *Genes chosen for validation by quantitative RT-PCR. (DOC) [file pone.0019909.s001.doc]

**Table S1.** **List of differentially expressed genes in natural Scrapie medulla oblongata and their associated functions based on GO analysis**. Only those genes with FC > 2 and a known GO term are shown. References for differentially expressed genes previously reported in other TSEs. *Genes chosen for validation by quantitative RT-PCR.

| **Function** | **Gene** | **Entrez gene ID** | **Fold change** | **Gene description** | **Citation** |
| --- | --- | --- | --- | --- | --- |
| **Carbohydrate binding** | [galM](http://www.ncbi.nlm.nih.gov/entrez/query.fcgi?db=gene&cmd=Retrieve&dopt=Graphics&list_uids=galM) | 616676 | -2,01 | Galactose mutarotase (aldose 1-epimerase) |  |
|  | [LOC654331](http://www.ncbi.nlm.nih.gov/entrez/query.fcgi?db=gene&cmd=Retrieve&dopt=Graphics&list_uids=LOC654331)* | 654331 | 8,32 | Pancreatitis-associated protein i* |  |
| **Carbohydrate transport** | [HK3](http://www.ncbi.nlm.nih.gov/entrez/query.fcgi?db=gene&cmd=Retrieve&dopt=Graphics&list_uids=HK3) | 3101 | 3,83 | Hexokinase 3 (white cell) |  |
|  | [SLC2A5](http://www.ncbi.nlm.nih.gov/entrez/query.fcgi?db=gene&cmd=Retrieve&dopt=Graphics&list_uids=SLC2A5) | 443507 | 2,06 | Solute carrier family 2 (facilitated glucose/fructose transporter), member 5 |  |
| **Cell adhesion** | [CD36](http://www.ncbi.nlm.nih.gov/entrez/query.fcgi?db=gene&cmd=Retrieve&dopt=Graphics&list_uids=CD36) | 281052 | 5,41 | Cd36 molecule (thrombospondin receptor) |  |
|  | [CD44](http://www.ncbi.nlm.nih.gov/entrez/query.fcgi?db=gene&cmd=Retrieve&dopt=Graphics&list_uids=CD44) | 281057 | 2,18 | Cd44 molecule (indian blood group) |  |
|  | [ITGB2](http://www.ncbi.nlm.nih.gov/entrez/query.fcgi?db=gene&cmd=Retrieve&dopt=Graphics&list_uids=ITGB2) | 494433 | 2,49 | Integrin, beta 2 (complement component 3 receptor 3 and 4 subunit) |  |
|  | [MPZL2](http://www.ncbi.nlm.nih.gov/entrez/query.fcgi?db=gene&cmd=Retrieve&dopt=Graphics&list_uids=MPZL2) | 540423 | -7,24 | Myelin protein zero-like 2 |  |
| **Cell growth regulation** | [IGFBP5](http://www.ncbi.nlm.nih.gov/entrez/query.fcgi?db=gene&cmd=Retrieve&dopt=Graphics&list_uids=IGFBP5) | 443133 | -3,03 | Insulin-like growth factor binding protein 5 | [30] |
|  | [MSTN](http://www.ncbi.nlm.nih.gov/entrez/query.fcgi?db=gene&cmd=Retrieve&dopt=Graphics&list_uids=MSTN) | 443449 | -2,21 | Myostatin |  |
|  | [kazald1](http://www.ncbi.nlm.nih.gov/entrez/query.fcgi?db=gene&cmd=Retrieve&dopt=Graphics&list_uids=kazald1) | 617445 | -2,15 | Kazal-type serine peptidase inhibitor domain 1 |  |
| **Cellular process** | [PRLH](http://www.ncbi.nlm.nih.gov/entrez/query.fcgi?db=gene&cmd=Retrieve&dopt=Graphics&list_uids=PRLH) | 443466 | -2,05 | Preproprolactin-releasing peptide |  |
|  | [NPY](http://www.ncbi.nlm.nih.gov/entrez/query.fcgi?db=gene&cmd=Retrieve&dopt=Graphics&list_uids=NPY) | 443508 | -3,11 | Neuropeptide y |  |
|  | [GREM1](http://www.ncbi.nlm.nih.gov/entrez/query.fcgi?db=gene&cmd=Retrieve&dopt=Graphics&list_uids=GREM1) | 539079 | -2,1 | Gremlin 1, cysteine knot superfamily, homolog |  |
|  | [MTNR1B](http://www.ncbi.nlm.nih.gov/entrez/query.fcgi?db=gene&cmd=Retrieve&dopt=Graphics&list_uids=MTNR1B)* | 100174786 | -7,65 | Melatonin receptor 1b* |  |
| **Cytokine activity** | [BMP3](http://www.ncbi.nlm.nih.gov/entrez/query.fcgi?db=gene&cmd=Retrieve&dopt=Graphics&list_uids=BMP3) | 651 | -3,52 | Bone morphogenetic protein 3 |  |
|  | [CXCL12](http://www.ncbi.nlm.nih.gov/entrez/query.fcgi?db=gene&cmd=Retrieve&dopt=Graphics&list_uids=CXCL12) | 613811 | -2,06 | Chemokine (c-x-c motif) ligand 12 (stromal cell-derived factor 1) |  |
| **Cytoskeletal protein binding** | [Mmp2](http://www.ncbi.nlm.nih.gov/entrez/query.fcgi?db=gene&cmd=Retrieve&dopt=Graphics&list_uids=Mmp2) | 282872 | -2,13 | Matrix metallopeptidase 2 (gelatinase a, 72kda gelatinase, 72kda type iv collagenase) |  |
|  | [capG](http://www.ncbi.nlm.nih.gov/entrez/query.fcgi?db=gene&cmd=Retrieve&dopt=Graphics&list_uids=capG) | 353121 | 2,24 | Capping protein (actin filament), gelsolin-like |  |
|  | [LOC100156254](http://www.ncbi.nlm.nih.gov/entrez/query.fcgi?db=gene&cmd=Retrieve&dopt=Graphics&list_uids=LOC100156254) | 100156254 | 2,55 | Similar to plastin-2 (l-plastin) (lymphocyte cytosolic protein 1) (lcp-1) (lc64p) |  |
| **DNA metabolic process** | [mgmt](http://www.ncbi.nlm.nih.gov/entrez/query.fcgi?db=gene&cmd=Retrieve&dopt=Graphics&list_uids=mgmt) | 616091 | -2,22 | O-6-methylguanine-dna methyltransferase |  |
| **Enzyme inhibitor activity** | [C3](http://www.ncbi.nlm.nih.gov/entrez/query.fcgi?db=gene&cmd=Retrieve&dopt=Graphics&list_uids=C3) | 280677 | 4,49 | Complement component 3 |  |
|  | [Serpinf1](http://www.ncbi.nlm.nih.gov/entrez/query.fcgi?db=gene&cmd=Retrieve&dopt=Graphics&list_uids=Serpinf1) | 281386 | -6,54 | Serpin peptidase inhibitor, clade f (alpha-2 antiplasmin, pigment epithelium derived factor), member 1 |  |
| **Glycoprotein binding** | [GPC3](http://www.ncbi.nlm.nih.gov/entrez/query.fcgi?db=gene&cmd=Retrieve&dopt=Graphics&list_uids=GPC3) | 615239 | -2,56 | Glypican 3 |  |
| **Immune system** | [CD14](http://www.ncbi.nlm.nih.gov/entrez/query.fcgi?db=gene&cmd=Retrieve&dopt=Graphics&list_uids=CD14) | 281048 | 2,24 | Cd14 molecule |  |
|  | [lap](http://www.ncbi.nlm.nih.gov/entrez/query.fcgi?db=gene&cmd=Retrieve&dopt=Graphics&list_uids=lap) | 403090 | 5,26 | Lingual antimicrobial peptide |  |
|  | [CD1E](http://www.ncbi.nlm.nih.gov/entrez/query.fcgi?db=gene&cmd=Retrieve&dopt=Graphics&list_uids=CD1E) | 510832 | 2,47 | Cd1e molecule |  |
|  | [CXCL13](http://www.ncbi.nlm.nih.gov/entrez/query.fcgi?db=gene&cmd=Retrieve&dopt=Graphics&list_uids=CXCL13) | 511674 | 2,32 | Chemokine (c-x-c motif) ligand 13 |  |
|  | [A2M](http://www.ncbi.nlm.nih.gov/entrez/query.fcgi?db=gene&cmd=Retrieve&dopt=Graphics&list_uids=A2M) | 513856 | 2,57 | Alpha-2-macroglobulin |  |
|  | [TLR7](http://www.ncbi.nlm.nih.gov/entrez/query.fcgi?db=gene&cmd=Retrieve&dopt=Graphics&list_uids=TLR7) | 554254 | 2,23 | Toll-like receptor 7 |  |
|  | [ifi30](http://www.ncbi.nlm.nih.gov/entrez/query.fcgi?db=gene&cmd=Retrieve&dopt=Graphics&list_uids=ifi30) | 615930 | 2,31 | Gamma-inducible protein 30 |  |
| **Ion binding** | [THBD](http://www.ncbi.nlm.nih.gov/entrez/query.fcgi?db=gene&cmd=Retrieve&dopt=Graphics&list_uids=THBD) | 281529 | -2,51 | Thrombomodulin |  |
|  | [MT2A](http://www.ncbi.nlm.nih.gov/gene/404070) | 404070 | 2,25 | Metallothionein 2a |  |
|  | [MT1A](http://www.ncbi.nlm.nih.gov/entrez/query.fcgi?db=gene&cmd=Retrieve&dopt=Graphics&list_uids=MT1A) | 404071 | 2,54 | Metallothionein 1e |  |
|  | [Egr1](http://www.ncbi.nlm.nih.gov/entrez/query.fcgi?db=gene&cmd=Retrieve&dopt=Graphics&list_uids=Egr1) | 407125 | -2,3 | Early growth response 1 |  |
|  | [CHI3L1](http://www.ncbi.nlm.nih.gov/entrez/query.fcgi?db=gene&cmd=Retrieve&dopt=Graphics&list_uids=CHI3L1) | 443279 | 3,83 | Chitinase 3-like 1 (cartilage glycoprotein-39) |  |
|  | [PTGS2](http://www.ncbi.nlm.nih.gov/entrez/query.fcgi?db=gene&cmd=Retrieve&dopt=Graphics&list_uids=PTGS2) | 443460 | -3,16 | Prostaglandin-endoperoxide synthase 2 (prostaglandin g/h synthase and cyclooxygenase) |  |
|  | [Egr1](http://www.ncbi.nlm.nih.gov/entrez/query.fcgi?db=gene&cmd=Retrieve&dopt=Graphics&list_uids=Egr1) | 443547 | -2,58 | Early growth response 1 | [30] |
|  | [GATA3](http://www.ncbi.nlm.nih.gov/entrez/query.fcgi?db=gene&cmd=Retrieve&dopt=Graphics&list_uids=GATA3) | 505169 | 5,83 | Gata binding protein 3 |  |
|  | [Ltbp1](http://www.ncbi.nlm.nih.gov/entrez/query.fcgi?db=gene&cmd=Retrieve&dopt=Graphics&list_uids=Ltbp1) | 510559 | -2,06 | Latent transforming growth factor beta binding protein 1 |  |
|  | [IDI1](http://www.ncbi.nlm.nih.gov/entrez/query.fcgi?db=gene&cmd=Retrieve&dopt=Graphics&list_uids=IDI1) | 514293 | 2,45 | Isopentenyl-diphosphate delta isomerase 1 |  |
|  | [Mmp12](http://www.ncbi.nlm.nih.gov/entrez/query.fcgi?db=gene&cmd=Retrieve&dopt=Graphics&list_uids=Mmp12) | 526981 | 2,14 | Matrix metallopeptidase 12 (macrophage elastase) |  |
|  | [nid1](http://www.ncbi.nlm.nih.gov/entrez/query.fcgi?db=gene&cmd=Retrieve&dopt=Graphics&list_uids=nid1) | 534319 | -4,15 | Nidogen 1 |  |
|  | [thbs4](http://www.ncbi.nlm.nih.gov/entrez/query.fcgi?db=gene&cmd=Retrieve&dopt=Graphics&list_uids=thbs4) | 541281 | -2,72 | Thrombospondin 4 |  |
|  | [PLS1](http://www.ncbi.nlm.nih.gov/entrez/query.fcgi?db=gene&cmd=Retrieve&dopt=Graphics&list_uids=PLS1) | 616560 | -2,92 | Plastin 1 (i isoform) |  |
|  | [S100A8](http://www.ncbi.nlm.nih.gov/entrez/query.fcgi?db=gene&cmd=Retrieve&dopt=Graphics&list_uids=S100A8) | 616818 | 2,16 | S100 calcium binding protein a8 |  |
| **Ion transport** | [KCNK2](http://www.ncbi.nlm.nih.gov/entrez/query.fcgi?db=gene&cmd=Retrieve&dopt=Graphics&list_uids=KCNK2) | 282590 | -2,69 | Potassium channel, subfamily k, member 2 |  |
|  | [CP](http://www.ncbi.nlm.nih.gov/entrez/query.fcgi?db=gene&cmd=Retrieve&dopt=Graphics&list_uids=CP) | 443053 | 3,7 | Ceruloplasmin |  |
|  | [CFTR](http://www.ncbi.nlm.nih.gov/entrez/query.fcgi?db=gene&cmd=Retrieve&dopt=Graphics&list_uids=CFTR) | 443347 | -3,16 | Cystic fibrosis transmembrane conductance regulator |  |
|  | [SLC11A1](http://www.ncbi.nlm.nih.gov/entrez/query.fcgi?db=gene&cmd=Retrieve&dopt=Graphics&list_uids=SLC11A1) | 443365 | 2,15 | Solute carrier family 11 (proton-coupled divalent metal ion transporters), member 1 |  |
|  | [ATP2A1](http://www.ncbi.nlm.nih.gov/entrez/query.fcgi?db=gene&cmd=Retrieve&dopt=Graphics&list_uids=ATP2A1) | 518117 | 2,58 | Atpase, ca++ transporting, cardiac muscle, fast twitch 1 | [30] |
| **Lipid binding** | [Ncf4](http://www.ncbi.nlm.nih.gov/entrez/query.fcgi?db=gene&cmd=Retrieve&dopt=Graphics&list_uids=Ncf4) | 507859 | 2,01 | Neutrophil cytosolic factor 4, 40kda |  |
| **Lipid metabolism** | [ACSM1](http://www.ncbi.nlm.nih.gov/entrez/query.fcgi?db=gene&cmd=Retrieve&dopt=Graphics&list_uids=ACSM1) | 282576 | -2,97 | Acyl-coa synthetase medium-chain family member 1 |  |
| **lipid transport** | [soat1](http://www.ncbi.nlm.nih.gov/entrez/query.fcgi?db=gene&cmd=Retrieve&dopt=Graphics&list_uids=soat1) | 504287 | 2,23 | Sterol o-acyltransferase 1 |  |
|  | [APOC4](http://www.ncbi.nlm.nih.gov/entrez/query.fcgi?db=gene&cmd=Retrieve&dopt=Graphics&list_uids=APOC4) | 618041 | 2,46 | Apolipoprotein c-iv |  |
| **Lysosomal** | [LAPTM4A](http://www.ncbi.nlm.nih.gov/entrez/query.fcgi?db=gene&cmd=Retrieve&dopt=Graphics&list_uids=LAPTM4A) | 404135 | -2,1 | Lysosomal protein transmembrane 4 alpha |  |
|  | [Laptm4b](http://www.ncbi.nlm.nih.gov/entrez/query.fcgi?db=gene&cmd=Retrieve&dopt=Graphics&list_uids=Laptm4b) | 404155 | -2,8 | Lysosomal protein transmembrane 4 beta |  |
|  | [CD68](http://www.ncbi.nlm.nih.gov/entrez/query.fcgi?db=gene&cmd=Retrieve&dopt=Graphics&list_uids=CD68) | 504960 | 2,09 | Cd68 molecule |  |
| **Nucleotide binding** | [FOS](http://www.ncbi.nlm.nih.gov/entrez/query.fcgi?db=gene&cmd=Retrieve&dopt=Graphics&list_uids=FOS) | 280795 | -2,66 | V-fos fbj murine osteosarcoma viral oncogene homolog |  |
|  | [GNA14](http://www.ncbi.nlm.nih.gov/entrez/query.fcgi?db=gene&cmd=Retrieve&dopt=Graphics&list_uids=GNA14) | 281789 | -3,29 | Guanine nucleotide binding protein (g protein), alpha 14 |  |
|  | [Hoxc6](http://www.ncbi.nlm.nih.gov/entrez/query.fcgi?db=gene&cmd=Retrieve&dopt=Graphics&list_uids=Hoxc6) | 443349 | -7,32 | Homeobox c6 |  |
|  | [HOXA5](http://www.ncbi.nlm.nih.gov/entrez/query.fcgi?db=gene&cmd=Retrieve&dopt=Graphics&list_uids=HOXA5) | 443358 | -9,76 | Homeobox a5 |  |
|  | [HOXA7](http://www.ncbi.nlm.nih.gov/entrez/query.fcgi?db=gene&cmd=Retrieve&dopt=Graphics&list_uids=HOXA7) | 443359 | -11,84 | Homeobox a7 |  |
|  | [UBTF](http://www.ncbi.nlm.nih.gov/entrez/query.fcgi?db=gene&cmd=Retrieve&dopt=Graphics&list_uids=UBTF) | 497012 | -2,08 | Upstream binding transcription factor, rna polymerase i |  |
|  | [PRKCD](http://www.ncbi.nlm.nih.gov/entrez/query.fcgi?db=gene&cmd=Retrieve&dopt=Graphics&list_uids=PRKCD) | 505708 | 2,13 | Protein kinase c, delta |  |
|  | [NR4A1](http://www.ncbi.nlm.nih.gov/entrez/query.fcgi?db=gene&cmd=Retrieve&dopt=Graphics&list_uids=NR4A1) | 528390 | -2,54 | Nuclear receptor subfamily 4, group a, member 1 |  |
|  | [RASL11B](http://www.ncbi.nlm.nih.gov/entrez/query.fcgi?db=gene&cmd=Retrieve&dopt=Graphics&list_uids=RASL11B) | 528446 | -2,57 | Ras-like, family 11, member b |  |
|  | [rab40b](http://www.ncbi.nlm.nih.gov/entrez/query.fcgi?db=gene&cmd=Retrieve&dopt=Graphics&list_uids=rab40b) | 534237 | -2,08 | Rab40b, member ras oncogene family |  |
|  | [TRM1L](http://www.ncbi.nlm.nih.gov/entrez/query.fcgi?db=gene&cmd=Retrieve&dopt=Graphics&list_uids=TRM1L) | 540872 | -2,41 | Hypothetical loc540872 |  |
| **Oxidation reduction** | [PLOD1](http://www.ncbi.nlm.nih.gov/entrez/query.fcgi?db=gene&cmd=Retrieve&dopt=Graphics&list_uids=PLOD1) | 281409 | 2,32 | Procollagen-lysine 1, 2-oxoglutarate 5-dioxygenase 1 |  |
|  | [aldh1a1](http://www.ncbi.nlm.nih.gov/entrez/query.fcgi?db=gene&cmd=Retrieve&dopt=Graphics&list_uids=aldh1a1) | 443343 | -2,19 | Aldehyde dehydrogenase 1 family, member a1 | [26,30,47] |
|  | [P4HA1](http://www.ncbi.nlm.nih.gov/entrez/query.fcgi?db=gene&cmd=Retrieve&dopt=Graphics&list_uids=P4HA1) | 518288 | -2,81 | Prolyl 4-hydroxylase, alpha polypeptide i |  |
| **Phosphate metabolic process** | [DUSP1](http://www.ncbi.nlm.nih.gov/entrez/query.fcgi?db=gene&cmd=Retrieve&dopt=Graphics&list_uids=DUSP1) | 539175 | -2,4 | Dual specificity phosphatase 1 |  |
| **Protein complex assembly** | [C1qtnf1](http://www.ncbi.nlm.nih.gov/entrez/query.fcgi?db=gene&cmd=Retrieve&dopt=Graphics&list_uids=C1qtnf1) | 511774 | -2,78 | C1q and tumor necrosis factor related protein 1 |  |
|  | [DOK2](http://www.ncbi.nlm.nih.gov/entrez/query.fcgi?db=gene&cmd=Retrieve&dopt=Graphics&list_uids=DOK2) | 514616 | 3,85 | Docking protein 2, 56kda |  |
| **Protein folding** | [FKBP5](http://www.ncbi.nlm.nih.gov/entrez/query.fcgi?db=gene&cmd=Retrieve&dopt=Graphics&list_uids=FKBP5) | 535704 | 2,92 | Fk506 binding protein 5 |  |
| **Protein transport** | [CHMP4B](http://www.ncbi.nlm.nih.gov/entrez/query.fcgi?db=gene&cmd=Retrieve&dopt=Graphics&list_uids=CHMP4B) | 616164 | -2,03 | Chromatin modifying protein 4b |  |
|  | [RAMP1](http://www.ncbi.nlm.nih.gov/entrez/query.fcgi?db=gene&cmd=Retrieve&dopt=Graphics&list_uids=RAMP1) | 617017 | -2,1 | Receptor (g protein-coupled) activity modifying protein 1 |  |
| **Proteolysis** | [HP](http://www.ncbi.nlm.nih.gov/entrez/query.fcgi?db=gene&cmd=Retrieve&dopt=Graphics&list_uids=HP) | 280692 | 4,62 | Haptoglobin |  |
|  | [Scpep1](http://www.ncbi.nlm.nih.gov/entrez/query.fcgi?db=gene&cmd=Retrieve&dopt=Graphics&list_uids=Scpep1) | 505054 | 2,19 | Serine carboxypeptidase 1 |  |
|  | [CTSH](http://www.ncbi.nlm.nih.gov/entrez/query.fcgi?db=gene&cmd=Retrieve&dopt=Graphics&list_uids=CTSH) | 510524 | 2,42 | Cathepsin h |  |
| **Signal transduction** | [WNT2B](http://www.ncbi.nlm.nih.gov/entrez/query.fcgi?db=gene&cmd=Retrieve&dopt=Graphics&list_uids=WNT2B) | 445420 | -2,49 | Wingless-type mmtv integration site family, member 2b |  |
|  | [SFRP4](http://www.ncbi.nlm.nih.gov/entrez/query.fcgi?db=gene&cmd=Retrieve&dopt=Graphics&list_uids=SFRP4) | 518004 | -3,81 | Secreted frizzled-related protein 4 |  |
| **Structural molecule activity** | [COL1A2](http://www.ncbi.nlm.nih.gov/entrez/query.fcgi?db=gene&cmd=Retrieve&dopt=Graphics&list_uids=COL1A2) | 282188 | -4,37 | Collagen, type i, alpha 2 |  |
|  | [col12a1](http://www.ncbi.nlm.nih.gov/entrez/query.fcgi?db=gene&cmd=Retrieve&dopt=Graphics&list_uids=col12a1) | 359712 | -2,76 | Collagen, type xii, alpha 1 |  |
|  | [LOC443079](http://www.ncbi.nlm.nih.gov/entrez/query.fcgi?db=gene&cmd=Retrieve&dopt=Graphics&list_uids=LOC443079) | 443079 | -4,66 | Type i keratin intermediate filament irsa1 |  |
|  | [GATA3](http://www.ncbi.nlm.nih.gov/entrez/query.fcgi?db=gene&cmd=Retrieve&dopt=Graphics&list_uids=GATA3) | 450296 | 3,63 | Gata binding protein 3 |  |
|  | [COL3A1](http://www.ncbi.nlm.nih.gov/entrez/query.fcgi?db=gene&cmd=Retrieve&dopt=Graphics&list_uids=COL3A1)* | 510833 | -4,05 | Collagen, type iii, alpha 1* |  |
|  | [NES](http://www.ncbi.nlm.nih.gov/entrez/query.fcgi?db=gene&cmd=Retrieve&dopt=Graphics&list_uids=NES) | 522383 | 2,55 | Nestin |  |
|  | [ANP32A](http://www.ncbi.nlm.nih.gov/entrez/query.fcgi?db=gene&cmd=Retrieve&dopt=Graphics&list_uids=ANP32A) | 538427 | -2,47 | Acidic (leucine-rich) nuclear phosphoprotein 32 family, member a |  |
|  | [K38](http://www.ncbi.nlm.nih.gov/entrez/query.fcgi?db=gene&cmd=Retrieve&dopt=Graphics&list_uids=K38) | 100141297 | -3,04 | Keratin 38 |  |
| **Transmembrane transport** | [AQP4](http://www.ncbi.nlm.nih.gov/entrez/query.fcgi?db=gene&cmd=Retrieve&dopt=Graphics&list_uids=AQP4) | 281008 | -2,09 | Aquaporin 4 |  |
|  | [AQP3](http://www.ncbi.nlm.nih.gov/entrez/query.fcgi?db=gene&cmd=Retrieve&dopt=Graphics&list_uids=AQP3) | 443047 | -2,76 | Aquaporin 3 |  |
| **Vitamin binding** | [BLG](http://www.ncbi.nlm.nih.gov/entrez/query.fcgi?db=gene&cmd=Retrieve&dopt=Graphics&list_uids=BLG) | 443385 | 11,68 | Beta-lactoglobulin |  |
| **Other** | [MFAP5](http://www.ncbi.nlm.nih.gov/entrez/query.fcgi?db=gene&cmd=Retrieve&dopt=Graphics&list_uids=MFAP5) | 281908 | -5,25 | Microfibrillar associated protein 5 |  |
|  | [DCN](http://www.ncbi.nlm.nih.gov/entrez/query.fcgi?db=gene&cmd=Retrieve&dopt=Graphics&list_uids=DCN) | 443048 | -2,96 | Decorin |  |
|  | [IGFBP-6](http://www.ncbi.nlm.nih.gov/entrez/query.fcgi?db=gene&cmd=Retrieve&dopt=Graphics&list_uids=IGFBP-6) | 443134 | -2,22 | Insulin-like growth factor binding protein 6 |  |
|  | [FOS](http://www.ncbi.nlm.nih.gov/entrez/query.fcgi?db=gene&cmd=Retrieve&dopt=Graphics&list_uids=FOS) | 443218 | -2,45 | C-fos protein |  |
|  | [Fst](http://www.ncbi.nlm.nih.gov/entrez/query.fcgi?db=gene&cmd=Retrieve&dopt=Graphics&list_uids=Fst) | 443323 | -2,15 | Follistatin |  |
|  | [SLC16A1](http://www.ncbi.nlm.nih.gov/entrez/query.fcgi?db=gene&cmd=Retrieve&dopt=Graphics&list_uids=SLC16A1) | 443456 | -2,34 | Solute carrier family 16, member 1 (monocarboxylic acid transporter 1) |  |
|  | [COL1A1](http://www.ncbi.nlm.nih.gov/entrez/query.fcgi?db=gene&cmd=Retrieve&dopt=Graphics&list_uids=COL1A1)* | 443483 | -2,67 | Collagen, type i, alpha 1* |  |
|  | [BMP5](http://www.ncbi.nlm.nih.gov/entrez/query.fcgi?db=gene&cmd=Retrieve&dopt=Graphics&list_uids=BMP5) | 462787 | -2,75 | Bone morphogenetic protein 5 |  |
|  | [WISP1](http://www.ncbi.nlm.nih.gov/entrez/query.fcgi?db=gene&cmd=Retrieve&dopt=Graphics&list_uids=WISP1) | 472862 | -3,58 | Wnt1 inducible signaling pathway protein 1 |  |
|  | [DENND2D](http://www.ncbi.nlm.nih.gov/entrez/query.fcgi?db=gene&cmd=Retrieve&dopt=Graphics&list_uids=DENND2D) | 504595 | 2,25 | Denn/madd domain containing 2d |  |
|  | [TREM2](http://www.ncbi.nlm.nih.gov/entrez/query.fcgi?db=gene&cmd=Retrieve&dopt=Graphics&list_uids=TREM2) | 506467 | 2,44 | Triggering receptor expressed on myeloid cells 2 |  |
|  | [NDE1](http://www.ncbi.nlm.nih.gov/entrez/query.fcgi?db=gene&cmd=Retrieve&dopt=Graphics&list_uids=NDE1) | 508088 | -2,05 | Nude nuclear distribution gene e homolog 1 (a. Nidulans) |  |
|  | [CCL26](http://www.ncbi.nlm.nih.gov/entrez/query.fcgi?db=gene&cmd=Retrieve&dopt=Graphics&list_uids=CCL26) | 508387 | 2,67 | Chemokine (c-c motif) ligand 26 |  |
|  | [LOC509574](http://www.ncbi.nlm.nih.gov/entrez/query.fcgi?db=gene&cmd=Retrieve&dopt=Graphics&list_uids=LOC509574) | 509574 | 2,05 | Similar to leukocyte immunoglobulin-like receptor |  |
|  | [C1QC](http://www.ncbi.nlm.nih.gov/entrez/query.fcgi?db=gene&cmd=Retrieve&dopt=Graphics&list_uids=C1QC) | 509968 | 2,42 | Complement component 1, q subcomponent, c chain |  |
|  | [CD84](http://www.ncbi.nlm.nih.gov/entrez/query.fcgi?db=gene&cmd=Retrieve&dopt=Graphics&list_uids=CD84) | 510910 | 2,61 | Cd84 molecule |  |
|  | [LOC511531](http://www.ncbi.nlm.nih.gov/entrez/query.fcgi?db=gene&cmd=Retrieve&dopt=Graphics&list_uids=LOC511531) | 511531 | -2,14 | Similar to guanylate binding protein 1 |  |
|  | [FBLN2](http://www.ncbi.nlm.nih.gov/entrez/query.fcgi?db=gene&cmd=Retrieve&dopt=Graphics&list_uids=FBLN2) | 511854 | -2,88 | Fibulin 2 |  |
|  | [CA13](http://www.ncbi.nlm.nih.gov/entrez/query.fcgi?db=gene&cmd=Retrieve&dopt=Graphics&list_uids=CA13) | 513850 | -2,14 | Carbonic anhydrase xiii |  |
|  | [pdgfrl](http://www.ncbi.nlm.nih.gov/entrez/query.fcgi?db=gene&cmd=Retrieve&dopt=Graphics&list_uids=pdgfrl) | 515017 | -2,37 | Platelet-derived growth factor receptor-like |  |
|  | [tp53i11](http://www.ncbi.nlm.nih.gov/entrez/query.fcgi?db=gene&cmd=Retrieve&dopt=Graphics&list_uids=tp53i11) | 515682 | -2,27 | Tumor protein p53 inducible protein 11 |  |
|  | [MMRN1](http://www.ncbi.nlm.nih.gov/entrez/query.fcgi?db=gene&cmd=Retrieve&dopt=Graphics&list_uids=MMRN1) | 516574 | 2,28 | Multimerin 1 |  |
|  | [SH3BP4](http://www.ncbi.nlm.nih.gov/entrez/query.fcgi?db=gene&cmd=Retrieve&dopt=Graphics&list_uids=SH3BP4) | 520462 | -2,06 | Sh3-domain binding protein 4 |  |
|  | [ttc21b](http://www.ncbi.nlm.nih.gov/entrez/query.fcgi?db=gene&cmd=Retrieve&dopt=Graphics&list_uids=ttc21b) | 527412 | -2,68 | Tetratricopeptide repeat domain 21b |  |
|  | [Sel1l3](http://www.ncbi.nlm.nih.gov/entrez/query.fcgi?db=gene&cmd=Retrieve&dopt=Graphics&list_uids=Sel1l3) | 535060 | -2,2 | Hypothetical loc535060 |  |
|  | [slc27a2](http://www.ncbi.nlm.nih.gov/entrez/query.fcgi?db=gene&cmd=Retrieve&dopt=Graphics&list_uids=slc27a2) | 535727 | -2,49 | Solute carrier family 27 (fatty acid transporter), member 2 |  |
|  | [Hs3st1](http://www.ncbi.nlm.nih.gov/entrez/query.fcgi?db=gene&cmd=Retrieve&dopt=Graphics&list_uids=Hs3st1) | 538691 | -3,01 | Heparan sulfate (glucosamine) 3-o-sulfotransferase 1 |  |
|  | [CAPN6](http://www.ncbi.nlm.nih.gov/entrez/query.fcgi?db=gene&cmd=Retrieve&dopt=Graphics&list_uids=CAPN6)* | 539360 | 5,07 | Calpain 6* |  |
|  | [BTG2](http://www.ncbi.nlm.nih.gov/entrez/query.fcgi?db=gene&cmd=Retrieve&dopt=Graphics&list_uids=BTG2) | 539364 | -2,15 | Btg family, member 2 |  |
|  | [Tmtc4](http://www.ncbi.nlm.nih.gov/entrez/query.fcgi?db=gene&cmd=Retrieve&dopt=Graphics&list_uids=Tmtc4) | 539972 | -2,1 | Transmembrane and tetratricopeptide repeat containing 4 |  |
|  | [ZNF428](http://www.ncbi.nlm.nih.gov/entrez/query.fcgi?db=gene&cmd=Retrieve&dopt=Graphics&list_uids=ZNF428) | 540173 | -2,27 | Zinc finger protein 428 |  |
|  | [C1H21ORF7](http://www.ncbi.nlm.nih.gov/entrez/query.fcgi?db=gene&cmd=Retrieve&dopt=Graphics&list_uids=C1H21ORF7) | 540879 | -2,5 | Chromosome 21 open reading frame 7 ortholog |  |
|  | [LOC554318](http://www.ncbi.nlm.nih.gov/entrez/query.fcgi?db=gene&cmd=Retrieve&dopt=Graphics&list_uids=LOC554318) | 554318 | 2,59 | Cd86/b7-2 costimulatory molecule |  |
|  | [LOC697375](http://www.ncbi.nlm.nih.gov/entrez/query.fcgi?db=gene&cmd=Retrieve&dopt=Graphics&list_uids=LOC697375) | 697375 | -3,33 | Similar to protease inhibitor 15 preproprotein |  |
|  | [ITGBL1](http://www.ncbi.nlm.nih.gov/entrez/query.fcgi?db=gene&cmd=Retrieve&dopt=Graphics&list_uids=ITGBL1) | 700965 | -3,67 | Integrin, beta-like 1 (with egf-like repeat domains) |  |
|  | [LOC705238](http://www.ncbi.nlm.nih.gov/entrez/query.fcgi?db=gene&cmd=Retrieve&dopt=Graphics&list_uids=LOC705238) | 705238 | -2,11 | Similar to b-cell cll/lymphoma 11b isoform 1 |  |
|  | [slc30a1](http://www.ncbi.nlm.nih.gov/entrez/query.fcgi?db=gene&cmd=Retrieve&dopt=Graphics&list_uids=slc30a1) | 745891 | 2,27 | Solute carrier family 30 (zinc transporter), member 1 |  |
|  | [MGC133880](http://www.ncbi.nlm.nih.gov/entrez/query.fcgi?db=gene&cmd=Retrieve&dopt=Graphics&list_uids=MGC133880) | 767965 | -2,96 | Hypothetical protein mgc133880 |  |
|  | [LOC777786](http://www.ncbi.nlm.nih.gov/entrez/query.fcgi?db=gene&cmd=Retrieve&dopt=Graphics&list_uids=LOC777786) | 777786 | 2,46 | Hypothetical protein loc777786 |  |
|  | [LOC780448](http://www.ncbi.nlm.nih.gov/entrez/query.fcgi?db=gene&cmd=Retrieve&dopt=Graphics&list_uids=LOC780448) | 780448 | -2,17 | Kappa opioid receptor |  |
|  | [NOD2](http://www.ncbi.nlm.nih.gov/entrez/query.fcgi?db=gene&cmd=Retrieve&dopt=Graphics&list_uids=NOD2) | 780465 | 2,23 | Nucleotide-binding oligomerization domain containing 2 |  |
|  | [GATA3](http://www.ncbi.nlm.nih.gov/entrez/query.fcgi?db=gene&cmd=Retrieve&dopt=Graphics&list_uids=GATA3) | 780483 | 3,69 | Gata binding protein 3 |  |
|  | [Hps3](http://www.ncbi.nlm.nih.gov/entrez/query.fcgi?db=gene&cmd=Retrieve&dopt=Graphics&list_uids=Hps3) | 783104 | 2,83 | Hermansky-pudlak syndrome 3 |  |
|  | [VN2R412P](http://www.ncbi.nlm.nih.gov/entrez/query.fcgi?db=gene&cmd=Retrieve&dopt=Graphics&list_uids=VN2R412P) | 784540 | 2,51 | Vomeronasal 2 receptor 412 pseudogene |  |
|  | [LOC785805](http://www.ncbi.nlm.nih.gov/entrez/query.fcgi?db=gene&cmd=Retrieve&dopt=Graphics&list_uids=LOC785805) | 785805 | -3,95 | Similar to collagen, type xxix, alpha 1 |  |
|  | [TNFRSF6B](http://www.ncbi.nlm.nih.gov/entrez/query.fcgi?db=gene&cmd=Retrieve&dopt=Graphics&list_uids=TNFRSF6B) | 789154 | 2,09 | Tumor necrosis factor receptor superfamily, member 6b, decoy |  |
|  | [SLAMF7](http://www.ncbi.nlm.nih.gov/entrez/query.fcgi?db=gene&cmd=Retrieve&dopt=Graphics&list_uids=SLAMF7) | 790164 | 2,83 | Slam family member 7 |  |
|  | [Ptx3](http://www.ncbi.nlm.nih.gov/entrez/query.fcgi?db=gene&cmd=Retrieve&dopt=Graphics&list_uids=Ptx3) | 100034672 | 4,07 | Pentraxin-related gene, rapidly induced by il-1 beta |  |
|  | [LOC100037673](http://www.ncbi.nlm.nih.gov/entrez/query.fcgi?db=gene&cmd=Retrieve&dopt=Graphics&list_uids=LOC100037673) | 100037673 | -3,72 | Cysteine-rich angiogenic inducer 61 |  |
|  | [LOC100037696](http://www.ncbi.nlm.nih.gov/entrez/query.fcgi?db=gene&cmd=Retrieve&dopt=Graphics&list_uids=LOC100037696)* | 100037696 | 6,79 | Galanin* |  |
|  | [PLIN2](http://www.ncbi.nlm.nih.gov/entrez/query.fcgi?db=gene&cmd=Retrieve&dopt=Graphics&list_uids=PLIN2) | 100125354 | 2,96 | Adipose differentiation-related protein |  |
|  | [LOC100125414](http://www.ncbi.nlm.nih.gov/entrez/query.fcgi?db=gene&cmd=Retrieve&dopt=Graphics&list_uids=LOC100125414) | 100125414 | 2,61 | Hypothetical protein loc100125414 |  |
|  | [LOC100153319](http://www.ncbi.nlm.nih.gov/entrez/query.fcgi?db=gene&cmd=Retrieve&dopt=Graphics&list_uids=LOC100153319) | 100153319 | -2,06 | Similar to discs, large homolog-associated protein 4 |  |
